# High-Content Monitoring of Drug Effects in a 3D Spheroid Model

**DOI:** 10.3389/fonc.2017.00293

**Published:** 2017-12-11

**Authors:** Frédérique Mittler, Patricia Obeïd, Anastasia V. Rulina, Vincent Haguet, Xavier Gidrol, Maxim Y. Balakirev

**Affiliations:** ^1^Université Grenoble Alpes, CEA, INSERM, BIG, BGE, Grenoble, France; ^2^Université Lyon 1, ENS de Lyon, INSERM, CNRS, CIRI, Lyon, France

**Keywords:** spheroids, 3D model, prostate cancer, MLN4924, HCS screening, drug discovery, ubiquitin, Nedd8

## Abstract

A recent decline in the discovery of novel medications challenges the widespread use of 2D monolayer cell assays in the drug discovery process. As a result, the need for more appropriate cellular models of human physiology and disease has renewed the interest in spheroid 3D culture as a pertinent model for drug screening. However, despite technological progress that has significantly simplified spheroid production and analysis, the seeming complexity of the 3D approach has delayed its adoption in many laboratories. The present report demonstrates that the use of a spheroid model may be straightforward and can provide information that is not directly available with a standard 2D approach. We describe a cost-efficient method that allows for the production of an array of uniform spheroids, their staining with vital dyes, real-time monitoring of drug effects, and an ATP-endpoint assay, all in the same 96-well U-bottom plate. To demonstrate the method performance, we analyzed the effect of the preclinical anticancer drug MLN4924 on spheroids formed by VCaP and LNCaP prostate cancer cells. The drug has different outcomes in these cell lines, varying from cell cycle arrest and protective dormancy to senescence and apoptosis. We demonstrate that by using high-content analysis of spheroid arrays, the effect of the drug can be described as a series of EC50 values that clearly dissect the cytostatic and cytotoxic drug actions. The method was further evaluated using four standard cancer chemotherapeutics with different mechanisms of action, and the effect of each drug is described as a unique multi-EC50 diagram. Once fully validated in a wider range of conditions, this method could be particularly valuable for phenotype-based drug discovery.

## Introduction

Past decades have witnessed a significant decline in the discovery of novel medications, which challenges the efficacy and even the validity of the modern drug discovery process (1–3). Among the identified causes of this failure is overreliance on the use of reductionist biological models in preclinical drug trials and, specifically, the use of immortalized cell lines cultured in an unnatural 2D setting (1). It is widely accepted that transformed cells growing in a monolayer on plastic dishes have little in common with the complex 3D multicellular organization found in living organisms. Awareness of this discrepancy has led to studies to find more appropriate cellular models to better represent human physiology and disease for drug screening. These models include long-known multicellular spheroids (4, 5) as well as more sophisticated recent developments, such as organotypic cultures and organoids (6–8), organ-on-a-chip (9, 10), and 3D-printed tissues (11). However, it should be noted that currently, few of these models can be implemented in classical drug discovery processes, which rely on high-throughput screening of thousands of chemical and biological entities in highly standardized assay conditions. Indeed, the increasing complexity of a model inevitably jeopardizes assay robustness, parallelization, integration, and data analysis, which are essential for standardization. It also boosts the cost of initial screening, although it can be worthwhile in subsequent clinical trials.

To date, the best compromise seems to be the use of spheroid-based models (4, 12, 13) because some of them can be easily standardized, and yet, they provide enough complexity to represent certain aspects of human tissues/tumors, such as 3D geometry; physical, chemical, and biological gradients; cell stratification; and functional differentiation. Spheroid culture is as old as cell culture itself (14, 15): the first pioneer that worked with cell cultures previously documented that “groups of cells round themselves off into little spheres” (16). Subsequent milestone works by Moscona in the 1950s (17) and Sutherland in the 1970s (18) led to the development of modern spheroid models as the preferred *in vitro* 3D platform for drug screening (4, 19–23).

Spheroids are formed by aggregation of cells into tight well-defined rounded objects. Many techniques have been developed to generate spheroids (12, 24), including growing cells in spinner flasks (25), in hanging drops (26), in levitation (27), or microgravity (28) within a natural (29, 30) or synthetic (31) polymer matrix and in liquid-overlay culture on agar- (32), agarose- (22), or polyHEMA-coated (33, 34) plates. In particular, agarose coating of standard microtiter plates creates concave-bottomed wells used to produce an array of individual spheroids and subsequent large-scale drug screening in a 3D format (20, 22, 23). However, because of reproducibility issues, in-lab coating is not always suitable for automated spheroid imaging (21).

To address the growing demand for highly standardized multiplex spheroid assays, several companies have developed spheroid-specific consumables, which include hanging-drop plates, ultra-low attachment (ULA) and similar plates, and micro/nanostructured plates and inserts (19). Among them, U-bottomed ULA plates have become the most popular due to the ease of use and compatibility with the majority of screening readouts [Table 1; (19)]. In particular, Corning-spheroid and InSphero GravityTRAP™ ULA plates facilitate the generation of highly uniform spheroids and their high-throughput/content analysis using bright-field and fluorescence microscopy. These plates are optimal for real-time monitoring of drug-induced changes in spheroid phenotypes.

**Table 1:** Commercial microtiter plates for 3D spheroid assays.

Microplate	References	Format	Bottom^1^	Color^2^	Surface	Company
Corning^®^ Ultra-Low Attachment (ULA)^3^	3474, 4520, 4515, 3830, 4516, 7007	96, 384	U	c, b	Grafted hydrogel	Corning Life Sciences
CELLSTAR^®^ Cell-Repellent Surface	650970, 651970	96	U/V	c	Grafted cell-repelling polymer	Greiner Bio-One
GravityTRAP™ ULA	ISP-09-001	96, 384	fV	c, b	Non-adhesive coating (NAC)	InSphero
CellCarrier^®^ ULA	6055330, 6055334	96	U	c	NAC	PerkinElmer
Nunclon™ Sphera™	174925, 174929	96	U	c	Sphera™ NAC	Thermo Fisher Scientific
PrimeSurface	MS-9096, MS-9384	96, 384	U/V/Sp	c, w	Grafted hydrophilic polymer	Sumitomo Bakelite
Nexcelom3D ULA	ULA-96U, ULA-384U	96, 384	U	c	NAC	Nexcelom Bioscience
Falcon^®^ 96 Non-Treated Assay	353910	96	U	c	Crystal-grade virgin polystyrene	Corning Life Sciences

*The microplate used in the present work is highlighted in yellow. (1) fV: wells with a V profile and a flat bottom. Sp: spindle shaped well; (2) c, clear; b, black; w, white; and (3) including Corning^®^-spheroid plates*.

However, it should be noted that ULA consumables remain rather expensive, and their use may not be necessary because similar quality results can be obtained with a simpler approach. Since the introduction of plasticware in cell culture, it has been known that a non-treated polystyrene surface is cell repelling (34–36), and early works from the Sutherland laboratory previously used this property for spheroid generation (37). By examining several commercially available U-bottomed non-treated plates, we found that the Falcon^®^ 96 Non-Treated Assay plate [henceforth “NTA plate,” Table 1] facilitates efficient spheroid assembly with a number of prostate cell lines. Moreover, excellent optical properties make them compatible with colorimetric, fluorescence, and even chemiluminescence assays as well as with various types of readout devices, such as standard and lens-free microscopes, high-content imaging systems, and microplate readers. As we demonstrate here, using these plates, the entire workflow from spheroid assembly, through real-time analysis of drug-induced phenotypic changes, to an ATP-endpoint assay can be performed on the same plate without spheroid transfer. This significantly simplifies the method and increases its performance.

One of the principal promises of the spheroid model in drug screening is that the more natural 3D setting will allow for more efficient identification of cancer-killing drugs or drugs potentially dangerous to normal cells. As a result, the most studied parameter of drug effects is cytotoxicity. Compared with cells in 2D monolayer culture, spheroids have an important advantage: their larger size. Thus, often, drug cytotoxicity can easily be followed over time by measuring the size and shape of spheroids (21, 38, 39). Furthermore, complex processes, such as tumor invasion and angiogenesis, can be modeled using spheroids, and the effect of drugs can be studied with simple bright-field microscopy (21, 40). Alternatively, the effect of drugs on cell composition, localization, and functional status within the spheroid can be analyzed using fluorescence microscopy, i.e., with immunofluorescence staining (41–43), expression of fluorescent proteins (44–46), and fluorescent probes (45). The latter is the most suited to a generic method for spheroid monitoring because it does not require spheroid fixation and can be applied to various types of cells.

Many fluorescent probes have been used in spheroid studies (Table 2), most often nuclear staining with Hoechst 33342 and viability measurements with Calcein-AM/Ethidium homodimer 1 (LIFE/DEAD assay, Thermo Scientific). Although cytotoxicity assays remain prevalent, several probes have been utilized for cell tracking or to examine cell function, reactive oxygen species (ROS) formation, mitochondrial activity, and important spheroid physiology parameters, such as oxygen gradient (Table 2).

**Table 2:** Fluorescent probes used for real-time spheroid monitoring.

Probe	Localization	Assay type	Ex/Em	Method	Reference
Hoechst 33342	Nucleus/DNA	Nuclei staining	350/461	HD, NAC, SFM, ULA	(20, 23, 47–53)
DRAQ5	Nucleus/DNA	Nuclei staining	647/665	HD, SFM	(54, 55)
SYTO11	Nucleus/DNA	Nuclei staining	508/527	HD, ULA	(52)
Propidium iodide	Nucleus/DNA	Viability/cytotoxicity	538/617	SFM, ULA	(51, 56)
SYTOX Green	Nucleus/DNA	Viability/cytotoxicity	488/523	NAC	(20, 23)
CellTox Green	Nucleus/DNA	Viability/cytotoxicity	485/530	ULA	(53)
Ethidium dimer	Nucleus/DNA	LIVE/DEAD^®^ assay	528/617	HD, NAC, SFM, ULA	(47, 48, 53, 57–60)
Calcein-AM	Cytoplasm	LIVE/DEAD^®^ assay	494/517	HD, NAC, SFM, ULA	(20, 47, 48, 53, 55–61)
DiOC18(3)	Membranes	Viability	484/501	NAC	(62)
Dihydroethidium	Nucleus/DNA	Cytotoxicity/ROS	518/605	HD, ULA	(52)
DCFDA	Whole cell	Cytotoxicity/ROS	495/529	SFM	(51)
MitoSOX Red	Mitochondria	Cytotoxicity/superoxide	510/580	ULA	(53)
MitoTrackers	Mitochondria	Cytotoxicity	Various	HD, NAC, ULA	(48, 49, 51–53, 63–66)
TMRE, TMRM	Mitochondria	Mitochondrial potential	540/580	HD, NAC, ULA	(52, 66)
Image-iT Assay	Mitochondria/other	Cytotoxicity	Various	ULA	(53)
NAD(P)H-Dye1	Mitochondria/other	Hypoxia/metabolism	537/561	NAC	(67)
CellTrackers	Whole cell	Cell labeling/tracking	Various	HD, NAC, SFM, ULA	(25, 50, 57, 59, 61, 65, 68, 69)
Vybrant DiD	Whole cell	Cell labeling/tracking	644/665	NAC	(20, 70)
Chlorobimane	Whole cell	Cytotoxicity/GSH	394/490	HD, ULA	(52)
ProSense^®^ 680	Whole cell	Proteolysis	680/700	ULA	(71)
Fluo-4-AM	Cytoplasm-Ca^2+^	Intracellular Ca^2+^	494/506	ULA	(52, 58)
HypoxiSense 680	Whole cell	Hypoxia/O_2_ gradient	680/700	ULA	(71)
Cyto-ID^®^ HRDR	Whole cell	Hypoxia/O_2_ gradient	596/670	HD, ULA	(72, 73)
NP SII-0.2+	Lysosome/other	Hypoxia/O_2_ gradient	620/760	NAC	(66)
NP NanO2	Cytoplasm	Hypoxia/O_2_ gradient	405/635	NAC	(66)
LOX-1	Whole cell	Hypoxia/O_2_ gradient	488/615	MSS, SFM	(60, 74, 75)
Ru-dpp	Whole cell	Hypoxia/O_2_ gradient	455/613	NAC	(62)
BCECF	Cytoplasm	pH gradient	490/535	NAC	(62)
NP T-probe	Cytoplasm	Temperature gradient	560/578	NAC	(66)
CaspGLOW Red	Cytoplasm	Apoptosis/caspases	540/570	NAC	(62)
Nucview 488	Nucleus/DNA	Apoptosis/caspases	504/534	HD	(76)
CellEvent	Nucleus/DNA	Apoptosis/caspases	511/533	NAC, NTA, ULA	(23, 47, 48, 77)
LysoTrackers	Lysosome/other	Cytotoxicity/apoptosis	Various	NAC, MSS, SFM	(66, 78–80)

*The probes used in the present work are highlighted in yellow*.

*HD, hanging drop; NAC, non-adhesive coating; NTA, non-treated assay; MSS, microstructured surface; SFM, spheroid forming medium/matrix; ULA, ultra-low attachment; ROS, reactive oxygen species*.

The primary difference between fluorescence staining of spheroids and monolayer culture staining is the reduced and uneven rate of dye transfer within the compact spheroid mass. This creates dye gradients and increases the time required to reach staining equilibrium. Dye uptake depends not only on the dye structure and type of spheroid but also on spheroid health and integrity. As a result, staining kinetics may be used as a phenotypic parameter to study the effect of drugs on spheroid state (see Results). However, due to dye cytotoxicity (Hoechst 33342), dye metabolism (Calcein-AM, ROS probes), or signal dilution (CellTrackers), in most published reports, the probes were added shortly before the endpoint assay (45).

In our method, we looked for fluorescent probes that can be used in combination with bright-field microscopy for long-term real-time spheroid imaging. For simple and efficient staining, we chose non-toxic CellEvent™ Caspase-3/7 Green and LysoTracker Deep Red probes that showed persistent spheroid fluorescence (>2 weeks) and provided information complementary to that obtained with bright-field microscopy (Table 2).

Apart from fluorescent staining, numerous standard metabolic assays have been applied to measure spheroid viability. Some of them, e.g., MTT tetrazolium reduction (81) and acid phosphatase assay [APH (22, 82)], use colorimetry, while others are based on fluorescence [Resazurin reduction (83, 84)] or chemiluminescence [ATP assay (85)]. It has been argued that gentle non-lytic assays, such as MTT or Resazurin reduction assays, may not be efficient with spheroids because of the incomplete probe penetration (86). In addition, a colorimetric APH assay has a limited sensitivity and requires spheroid transfer (22). Today, the most robust metabolic endpoint assay seems to be the measurement of spheroid ATP content *via* chemiluminescence. Efficient ATP extraction is achieved by harshening (compared with monolayer culture) the lysis conditions or by using commercial 3D-specific reagents (38, 85). The chemiluminescence method has excellent sensitivity, and as we demonstrate in the present report, the ATP content of a single spheroid can be quantified reliably in the same U-bottomed plate after completion of phenotypic analysis.

To demonstrate the method performance, we analyzed the effect of the preclinical anticancer drug MLN4924 (henceforth “MLN”) on spheroids formed by VCaP and LNCaP prostate cancer cells. MLN, an inhibitor of Nedd8-activating enzyme (NAE), blocks intracellular proteolysis mediated by cullin-RING E3 ligases (87). MLN is currently being evaluated in clinical trials for the treatment of hematologic malignancies and solid tumors (88, 89). Different cancer cells have different sensitivities to MLN, and as we have shown recently (77), MLN treatment has complex outcomes that vary from cell cycle arrest and protective dormancy to senescence and apoptosis. Here, we show that the complexity of the effects of MLN as well as of other cancer therapeutics with different mechanisms of action (MOAs) can be addressed using the 3D spheroid method.

## Materials and Methods

### Materials

Falcon^®^ 96 Well Clear Round Bottom Non-Treated Assay plates (NTA plates, Corning #353910) and White 96 Well Transparent Bottom CELLSTAR^®^ plates (white plates, Greiner Bio-One #655088) were purchased from Dominique Dutscher (Grenoble, France). Universal polystyrene lids (Thermo Fisher Scientific #5500), Dulbecco’s modified Eagle’s medium (DMEM, Gibco #41966), Roswell Park Memorial Institute medium (RPMI1640, Gibco #61870), Keratinocyte Serum Free Medium (K-SFM) kit supplied with bovine pituitary extract (BPE), and human epidermal growth factor (EGF) (Gibco #17005-042), Dulbecco’s phosphate-buffered saline (PBS) without calcium or magnesium (Gibco #14190), 0.05% trypsin–EDTA (Gibco #25300054), 10 kU/mL penicillin–streptomycin (Gibco #15140122), CellEvent Caspase-3/7 Green Reagent (Invitrogen #C10423), and LysoTracker Deep Red (Invitrogen #L12492) were obtained from Thermo Fisher Scientific (Courtaboeuf, France). Fetal bovine serum (FBS, PAN Biotech #P30-3302) was purchased from Dominique Dutscher (Grenoble, France). ViaLight™ Plus Cell Proliferation and Cytotoxicity BioAssay Kit (Lonza #LT07-121) were obtained from Lonza (Basel, Switzerland). For chemotherapy drugs, MLN4924 (MLN, MedChemExpress #LSG790) was purchased from Interchim (Montluçon, France), cisplatin (Cayman Chemical #CAYM13119-250) from VWR International (Fontenay-sous-Bois, France), docetaxel (Acros Organics, #15316097) from Thermo Fisher Scientific, Etoposide (Aldrich, #E1383) from Sigma-Aldrich (Saint-Quentin-Fallavier, France), and ARN-509 (Adooq Bioscience, #A11923-10) from CliniSciences (Nanterre, France).

### Cell Lines

The VCaP (androgen receptor-positive, androgen-sensitive, containing *TMPRSS-ERG* translocation, p53-R248W, PTEN-wt; ATCC #CRL-2876), LNCaP (androgen receptor-positive, androgen dependent, p53-wt, PTEN-mut; ATCC #1740), PC3 (androgen receptor-negative, androgen independent, p53-null, PTEN-null; ATCC #CRL-1435), DU145 (androgen receptor-negative, androgen-independent, p53-P223L-V274F, PTEN-wt; ATCC #HTB-81), and RWPE-1 (HPV18-immortalized normal adult prostate epithelial cells; ATCC #CRL-11609) cell lines were purchased from American Type Culture Collection (ATCC). The DuCaP cell line (similar origin as VCaP) was kindly provided by Prof. Jack Schalken from the Radboud University Nijmegen Medical Center, who originally received them from Kenneth J. Pienta, MD, Director of Research at The Brady Urological Institute, Baltimore, where this cell line was created (90). The PNT2 cell line (SV40-immortalized normal adult prostate epithelial cells; ECACC #95012613-1VL) was purchased from the European Collection of Authenticated Cell Cultures (ECACC).

Cell lines were tested on a semester basis for AR, PSA, SLC45A3, and ERG using quantitative RT-PCR. VCaP and DU145 cells were cultured in DMEM containing 10% FBS and 1% penicillin–streptomycin (complete growth medium). LNCaP, PC3, DuCaP, and PNT2 cells were cultured in RPMI1640 with the same supplements. RWPE-1 cells were cultured in K-SFM containing 0.05 mg/mL BPE, 5 ng/mL EGF, and 1% penicillin–streptomycin. The cells were grown in an incubator at 37°C, 5% CO_2_, and 95% humidity. For different passages, the cells were washed twice with PBS followed by the addition of trypsin–EDTA and incubation for 3–10 min depending on the cell line. Subculture was performed depending on the density of the cells. Typically, for VCaP cells, subculture was performed once per week, at 1/2 dilution; LNCaP cells were divided twice per week, at 1/5 dilution.

### Spheroid Assembly

**Figure 1:**
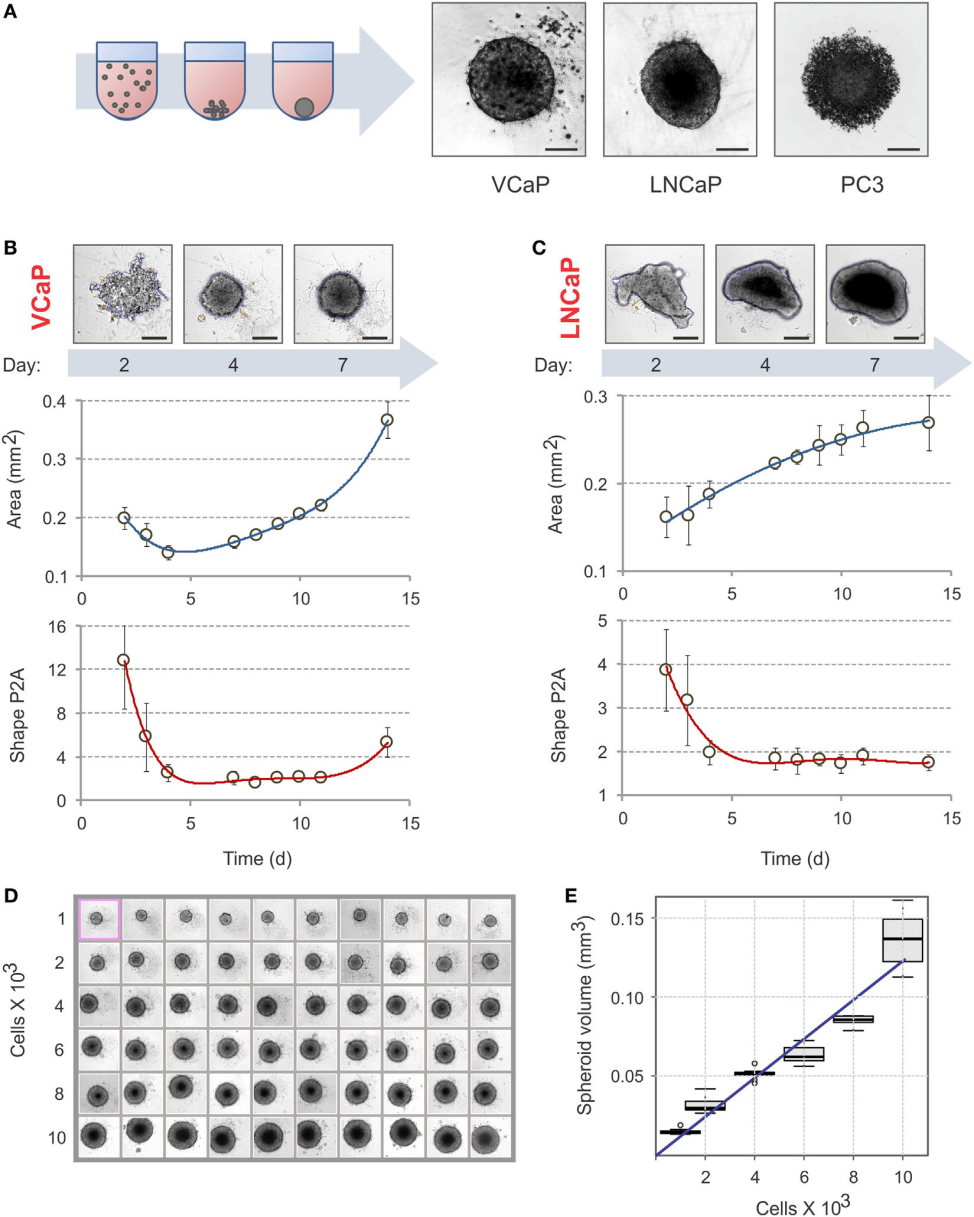
Spheroid assembly in U-bottom, 96-well NTA plates. **(A)** Schematic diagram of spheroid assembly. Representative images on the right show the aggregates formed by VCaP (4,000 cells seeded), LNCaP (1,000 cells seeded), and PC3 (500 cells seeded) cells in 7 days. The images were acquired with a Zeiss Observer Z1 microscope. Scale bar, 200 µm. For real-time observation of VCaP spheroid assembly, see Movie S1 in Supplementary Material. **(B,C)** Kinetics of spheroid assembly. Spheroids were formed with **(B)** 2,000 VCaP cells and **(C)** 500 LNCaP cells. The images were acquired with a CellInsight NXT HCS platform, and the aggregate “Area” and “Shape P2A” (roundness) metrics were calculated using a Morphology.V4 application. Scale bar, 200 µm. The plots show the data analyzed with “R” statistics software (mean ± SD, 24 spheroids per cell line). **(D)** Uniformity of VCaP spheroid assembly. Representative CellInsight images of spheroids formed by varying numbers of VCaP cells over 7 days. The field of view is 896.6 µm × 896.6 µm. **(E)** The box plots show the corresponding VCaP spheroid volumes estimated with Morphology.V4 and analyzed with R software. The slope of the linear trend line corresponds to ~12 pL of occupied volume per seeded cell.

Cells were grown in appropriate complete growth medium for at least 3 days in standard T75 flasks to reach approximately 70% confluence. For reproducible results, cells of a similar passage number (1 < *N* < 15) were always used. To prepare the cell suspension, a cell monolayer was washed with 10 mL of PBS and treated with 1.5 mL of a trypsin–EDTA solution for 5 min at 37°C. Trypsin was then neutralized with 8 mL of complete growth medium, and the cell suspension was centrifuged at 400 × *g* for 5 min. After supernatant removal, the cell pellet was resuspended in 1–3 mL of complete growth medium, and the cell density was determined using a Scepter™ Cell Counter (Merck Millipore). Note that it is critical to ensure that the cell suspension is homogeneous (single cells) and does not contain aggregates, because this determines the size and uniformity of spheroids. The optimal cell density must be determined in preliminary spheroid generation tests (see Figure 1C, for example).

The suspension was then diluted with complete growth medium to obtain the final density and dispensed (200 μl/well) into 96-well NTA plates. To reduce the effect of uneven evaporation on spheroid formation, the edge wells of the plates were not used and filled with PBS. This resulted in 6 × 10 = 60 wells per plate to analyze. The plates were covered with the universal polystyrene lids and kept in an incubator (37°C, 5% CO_2_, 95% humidity) for at least 2 days before imaging. During this period, it is important not to disturb the plates as this can displace spheroids from the center of the well. In addition, although previous reports (70, 83, 91) have shown that mild centrifugation can promote single spheroid assembly, no significant effect of centrifugation was observed with VCaP and LNCaP cells.

Finally, the analysis of spheroid assembly was conducted immediately after the cell suspension addition when performed on a video microscope (Zeiss Axiovert 200 M) equipped with a cell culture chamber or on a lens-free microscope [VideoCell (92)] within the cell culture incubator.

### Analysis of Spheroid Assembly

After 2 days of spheroid formation, the plates were scanned once a day on a CellInsight NXT High Content Screening Platform (Thermo Scientific) equipped with a 10× objective. The acquisition parameters were set using the instrument software HCS Studio 6.5.0. The bright-field channel was used for autofocusing, and the exposure time was set within the 25–35% saturation range according to the HCS Studio recommendations. Acquisition of “one field of view per well” was sufficient for the majority of spheroid measurements and significantly reduced the time required for plate analysis. Using 4 × 4 binning, an 896.6 µm × 896.6 µm field of view was converted into a 552 px × 552 px image. With these settings, the scan of a 96-well plate took approximately 5 min. The images were saved automatically and could be analyzed later.

Image analysis was performed with the instrument software HCS Studio 6.5.0—Morphology.V4. Up to 20 different “Cell-selected features” were chosen for Channel 1. Then, the bright-field images were imported as “Channel 1-Object.” This generated the object masks for each image. The masks were further adjusted by imposing the size range for the counted objects (“Object selection” > 1,000 px^2^ for VCaP, and >3,000 px^2^ for LNCaP) and “Cell-selected features” were calculated for each of the adjusted masks. The object “Area” and “Shape P2A” were the most informative morphometric parameters (see Results). Shape P2A = (Perimeter)^2^/4π × (Area) represents the object roundness and is equal to 1 for a perfect circle; for less spherical objects, P2A becomes larger than 1. Note that, apart from these features, other calculated parameters, such as object “CircDiameter,” “Sphere Volume,” and “Aspect ratio LWR” were also useful for evaluating spheroid morphology. Next, a table of all calculated features was generated as a .csv file using the “Cellomics-View” application. The data were imported into “R” statistical software to create a fully annotated .csv data file by fusing with the “Experimental Design” table. The mean, median, SD, and quartile values were calculated with “R” software.

For manual acquisition, the images from each well were recorded using an inverted microscope (Zeiss Observer Z1). A 10× objective was primarily used, switching to a 5× objective when an object area was too large. The entire acquisition took approximately 30 min for a 96-well plate. To analyze the images taken on standard and video microscopes, an in-lab ImageJ macro was used. Alternatively, open-source tools, e.g., based on MATLAB (39), ImageJ (83), or Fiji (93), could also be utilized to produce object masks and calculate object area and perimeter.

### Spheroid Treatment with MLN4924

The spheroids were formed following the protocol described earlier by incubating 2,000 cells for 7 days (VCaP) or 500 cells for 4 days (LNCaP) in NTA plates. Three replicate plates were prepared per experiment. MLN solutions were prepared immediately before use by diluting 2 mM DMSO stock solution in complete growth medium to a 2× final concentration. The drug solution of the highest concentration was made first and used to prepare all drug dilutions of the series. To avoid the effect of DMSO on spheroid growth, it is critical to ensure that the highest DMSO concentration in spheroid medium does not exceed 0.5%, which corresponds to 1% in drug solutions. Using “6 × 10 wells per plate” and “six spheroids per condition” experimental formats, nine drug concentrations and the DMSO vehicle control could be tested with one NTA plate. This corresponded to 600 µL total volume per plate for each drug dilution.

For apoptosis analysis, CellEvent was added to each of the prepared drug solutions to obtain a 2 µM concentration. Then, 100 µL of spheroid medium was carefully removed from each well (trying not to displace the spheroids from the center of the well) and replaced with 100 µL of CellEvent-drug solution. The plates were kept in an incubator (37°C, 5% CO_2_, 95% humidity), and image acquisition was performed once a day.

For viability measurements, a 0.4 µM solution of LysoTracker Deep Red dye was prepared just before use by diluting 1 mM DMSO stock solution in complete growth medium. Then, 10 µL of this solution was added to each well, and imaging was performed at time points defined in the “Experimental Design” table.

### Monitoring MLN Effect

The effect of MLN on spheroids was followed using a CellInsight NXT High Content Screening Platform as described earlier. The acquisition parameters were as follows: bright field for Channel 1, CellEvent fluorescence (FITC filter set used) for Channel 2, and LysoTracker Deep Red fluorescence (Cy5 filter set used) for Channel 3. Autofocus was performed in bright field (Channel 1), and the plates were scanned using “one field of view per well” acquisition with 4 × 4 binning. With these settings, the scan of a 96-well plate usually took less than 7 min.

The image analysis was performed using HCS Studio 6.5.0—Morphology.V4 software as described earlier. Up to 30 different “Cell-selected features” were measured for Channels 1–3. The bright-field images were imported as “Channel 1—Object,” CellEvent images as “Channel 2—Member,” and LysoTracker Deep Red images as “Channel 3—Spot/Fiber.” The corresponding masks were generated for each image, and “Cell-selected features” were calculated for each mask. The preferred parameters used for fluorescence analysis of the drug effects were fluorescence “Area,” “Total Intensity,” and “Average Intensity.” The data were analyzed using “R” software.

For manual acquisition, the images were recorded on the inverted microscope with a 10× (5×) objective using bright field, FITC, and Cy5 channels. The analysis was performed using an in-lab ImageJ macro.

### ATP-Based Endpoint Assay

Spheroid viability was analyzed by measuring ATP with a ViaLight™ Plus Cell Proliferation and Cytotoxicity BioAssay Kit (Lonza). The manufacturer’s protocol was applied with some modifications. Thus, 100 µL of spheroid medium was carefully removed from each spheroid-containing well of the NTA plate, and 50 µL of cell lysis reagent was added. The plate was agitated on an orbital shaker for 20 min at room temperature. Note that this protocol has been optimized for VCaP and LNCaP spheroids. For other cell types, the optimal lysis time must be determined in preliminary tests by measuring the efficiency of ATP extraction (using chemiluminescence).

Then, 100 µL of room-temperature ATP monitoring reagent plus (AMR plus) was added to each well, and the plate was incubated for 2 min at room temperature. Blank solution was prepared by adding cell lysis reagent and AMR plus to complete growth medium. Luminescence was measured on a GloMax^®^-Multi Detection System (Promega) with a 1 s integration time. Optionally and when higher sensitivity is required, the samples can be transferred into a white plate with a flat transparent bottom (Grenier Bio-One) and remeasured. This may result in an up to threefold increase in signal intensity.

Cell viability in monolayer cultures was measured according to the manufacturer’s protocol.

### Monitoring the Effect of Chemotherapy Drugs

The effect of chemotherapy drugs (cisplatin, docetaxel, etoposide, and ARN-509) on VCaP and LNCaP spheroids was examined following the protocol described earlier for MLN. Drug stock solutions were prepared in DMSO, with the exception of cisplatin, for which DMF was used (94). Drug dilutions used to generate dose–response curves are given in Table S1 in Supplementary Material.

## Results

### Spheroid Assembly

Because cell attachment is inhibited in NTA plates, the cells clump together and form 3D spheroids. The U-profile of the wells ensures that only one spheroid per well is formed (Figure 1A; Movie S1 in Supplementary Material). As with ULA plates (21), not all cells form spheroids under these conditions. For instance, VCaP and LNCaP prostate cancer cell lines form spheroids while PC3 cells produce only loose aggregates [Figure 1A; see also Ref. (21, 33, 95)]. In some cases, modification of the cell culture medium composition could promote spheroid formation in NTA plates. Thus, among the seven prostate cancer cell lines examined in the present work, the majority produced individual spheroids after brief optimization of the culture conditions (Figure S1 in Supplementary Material). Two cell lines, DU145 and PNT2, formed multiple non-uniform spheroid-like aggregates (Figure S1 in Supplementary Material). Because VCaP and LNCaP cells reproducibly form spheroids and have different sensitivities to MLN in monolayer culture (77), we focused our study on these cell lines.

To avoid drug interference with spheroid assembly, spheroids must be well formed before drug treatment. The criteria used in the literature are rather visual and arbitrary (21, 31). We therefore decided to define a measurable parameter of spheroid assembly. Spheroid formation was followed over time with a CellInsight NXT HCS platform and analyzed with the instrument software (Figures 1B,C). We found that “Area” and “Shape P2A” are the best direct morphometric parameters to characterize spheroid assembly and growth. For slow-proliferating (in 2D) cells, such as VCaP cells, the minimum of the “Area” curve may represent a characteristic time of spheroid assembly (Figure 1B). However, for the faster growing LNCaP cells, the “Area” occupied by the aggregate steadily increased (Figure 1C). The best parameter for both cell lines appeared to be “Shape P2A”: when it attains the minimum, the aggregates are considered round and spheroid (Figures 1B,C). Using this parameter, a period of 4–7 days was found to be optimal for spheroid assembly. It should be noted that when measuring spheroid assembly, particular attention must be paid to the composition of the culture medium. Thus, we found that small changes in medium FBS, e.g., different concentrations, suppliers, and even lots, may significantly influence spheroid assembly. In addition, charcoal stripping of the serum reduced the rate of spheroid formation by both VCaP and LNCaP cells (Figure S1 in Supplementary Material). Therefore, it seems that spheroid assembly in NTA plates is, at least partially, an active cellular process that depends on specific cues within the culture medium.

The NTA plates ensured the formation of highly uniform spheroids (Figure 1D). Increasing the density of cell seeding resulted in a linear increase in spheroid volume. For VCaP cells, the slope of the trendline corresponded to approximately 12 pL of occupied volume per seeded cell (Figure 1E), which fell within the range of 5–20 pL/cell estimated for other spheroids described in the literature (21, 47, 83). This significantly exceeds the size of a VCaP cell (1.3 pL) measured with a Scepter™ Cell Counter, suggesting that cells undergo multiplication upon spheroid assembly. This could also indicate the presence of some extracellular space within the spheroids.

The optimal conditions required to form spheroids of approximately 400 µm in diameter (21, 31, 83) were determined to be 2,000 cells/well for 7 days with VCaP cells. Because the fast-growing LNCaP spheroids rapidly developed a hypoxic core that interfered with phenotypic measurements (see below), 500 cells/well and a duration of 4 days were used to form LNCaP spheroids.

### Effect of MLN on Spheroid Growth, Morphology, and Apoptosis

Having established the optimal conditions for spheroid assembly, we examined the effect of MLN on preformed VCaP and LNCaP spheroids. Of note, in monolayer culture, these cell lines display a marked difference in MLN sensitivity: IC50 of 500 and 50 nM for VCaP and LNCaP cells, respectively.

**Figure 2:**
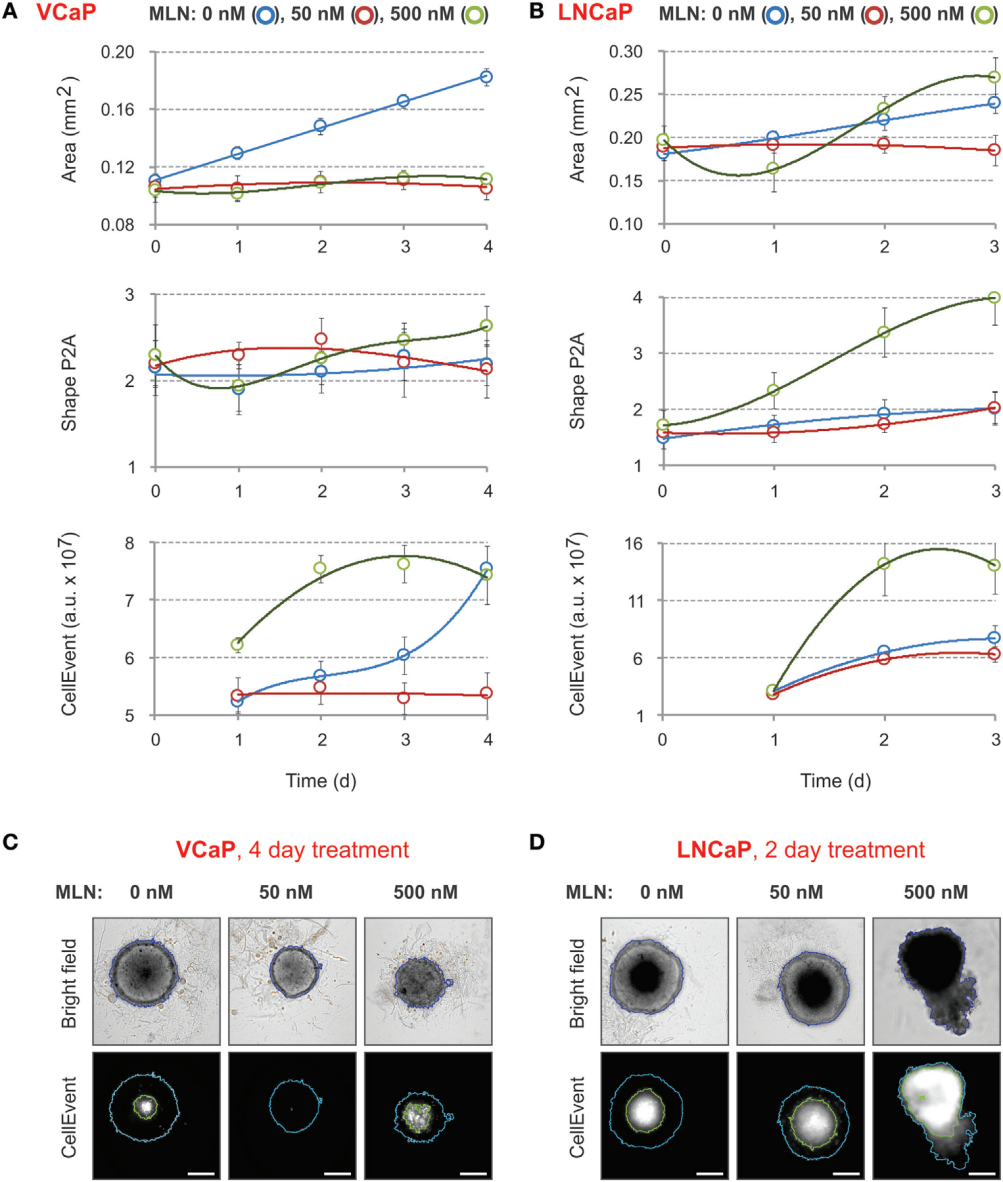
Effect of MLN on spheroid growth, morphology, and apoptosis. **(A,B)** Spheroids were preformed **(A)** for 7 days with 2,000 VCaP cells and **(B)** for 4 days with 500 LNCaP cells and treated (at Time = 0) with the indicated concentrations of MLN. CellEvent^®^ Caspase-3/7 Green Detection Reagent (1 µM final) was added at the same time. The images were acquired once a day with the CellInsight NXT HCS platform. Spheroid “Area” and “Shape P2A” [blue segmentation, **(C,D)**] as well as CellEvent fluorescence (FITC filter set) and fluorescence area [green segmentation, **(C,D)**] were measured using a Morphology.V4 application. The plots show the changes in spheroid morphology and CellEvent fluorescence analyzed with “R” software (mean ± SD, six spheroids per condition). **(C,D)** Representative images of **(C)** VCaP and **(D)** LNCaP spheroids were acquired with the CellInsight NXT HCS platform at day 4 (VCaP) or at day 2 (LNCaP) of the treatment. Scale bar, 200 µm.

First, the time course of changes in spheroid size and shape induced by 50 and 500 nM of MLN was analyzed (Figures 2A,B). For VCaP cells, at both concentrations, the drug arrested the growth of spheroids without affecting their roundness (Figure 2A, “Area” vs “Shape P2A”). By contrast, automated analysis of LNCaP spheroid growth was not conclusive (Figure 2B, “Area”). Visual examination revealed that at 500 nM the drug caused physical disruption of LNCaP spheroids, resulting in an unexpected increase in apparent spheroid size (Figure 2D, “Bright field”). The disruption starts on the second day of MLN treatment and is easily detected automatically by measuring spheroid roundness (Figure 2B, “Shape P2A”).

These results demonstrate that within the given time-frame simple bright-field analysis of spheroid morphology is not sufficient to characterize the effect of MLN. Indeed, it does not distinguish between two very different drug concentrations in the case of VCaP spheroids, whereas in LNCaP spheroids, it does not reliably detect the toxic effect of 50 nM MLN that corresponds to the IC50 value in monolayer culture. More prolonged drug treatment can partially help but requires reevaluation of culture conditions to prevent spheroid overgrowth.

Because MLN induces apoptosis in prostate cancer cells (77), we measured the kinetics of apoptosis induction using CellEvent, a fluorogenic caspase-3/7 substrate (23, 77) (Figures 2C,D, “CellEvent”). The reagent was added to spheroids at the same time as MLN. Drug-induced apoptosis was readily detected in both VCaP and LNCaP spheroids starting from the second day of spheroid treatment with 500 nM MLN (Figures 2A,B, “CellEvent”). In addition, independently of the treatment, all spheroids larger than approximately 450 µm in diameter showed a CellEvent-positive central region consistent with apoptosis induction within the nutrient deprived spheroid core (23, 62, 77). When this signal is so strong that it interferes with the drug cytotoxicity assay, as in LNCaP spheroids (Figure 2D), the measurement of the “fluorescence area” and normalization of the CellEvent signal to the spheroid area may help to distinguish the drug-specific effect. Thus, although the measurement of CellEvent “total intensity” did not distinguish between control and 50 nM MLN conditions (Figure 2B, “CellEvent”), some increase in “fluorescence area” was observed upon drug treatment (Figure 2D). Finally, and consistent with our previous observation (77), VCaP spheroids growth arrested with 50 nM MLN showed negligible apoptosis, even compared with control conditions (Figure 2C, “CellEvent”).

Compared with the morphometric parameters, the use of CellEvent provides complementary information regarding how the drug induces apoptosis within spheroids. Notably, for all tested concentrations (<2 µM), CellEvent was non-toxic to prostate cancer spheroids (did not change morphometric parameters or ATP content) and ensured a measurable and informative signal for more than 2 weeks of spheroid culture.

### Spheroid Staining with LysoTracker Deep Red

**Figure 3:**
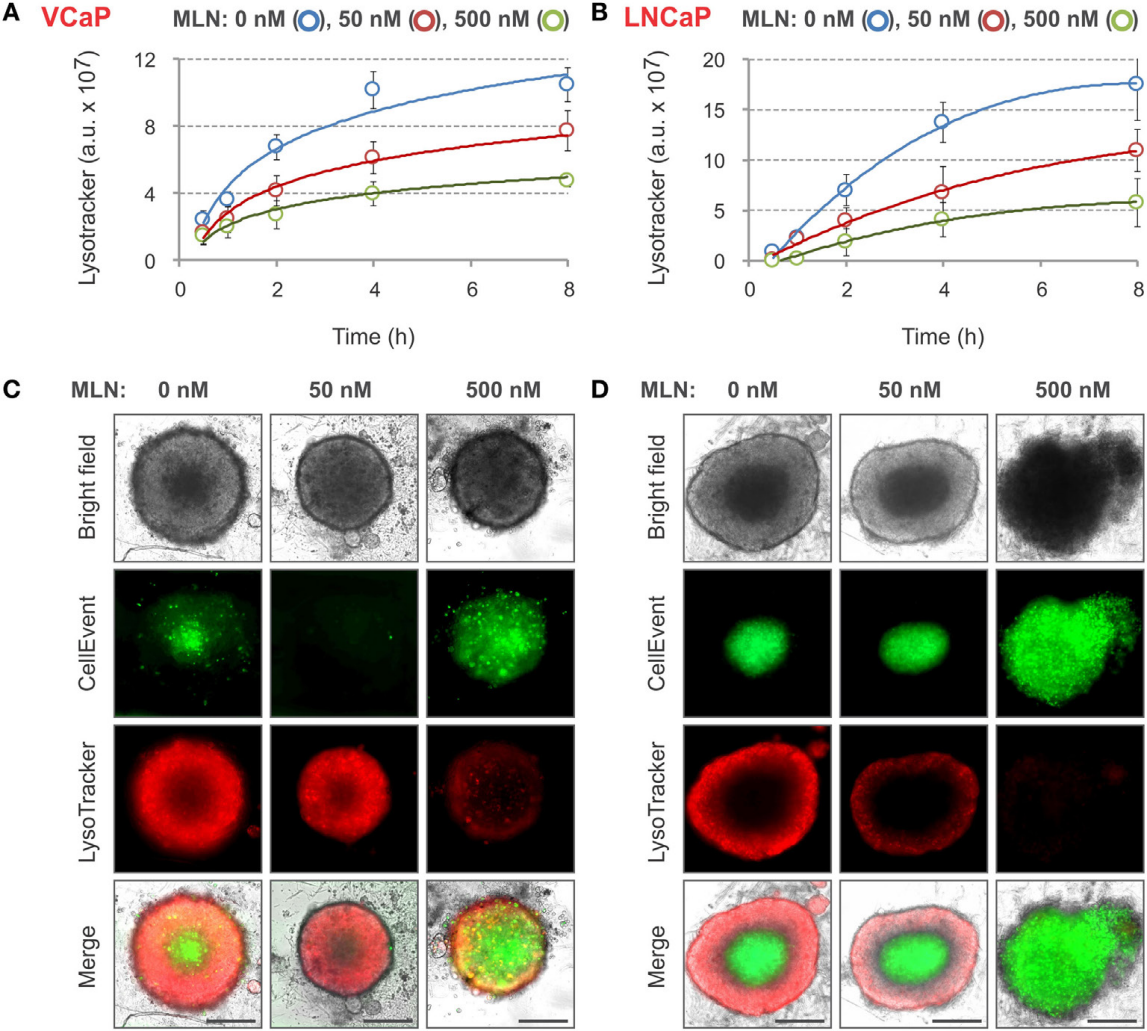
LysoTracker Deep Red staining of MLN-treated spheroids. **(A,B)** Spheroids were preformed **(A)** for 7 days with 2,000 VCaP cells and **(B)** for 4 days with 500 LNCaP cells and treated for 4 days (VCaP) or 2 days (LNCaP) with MLN in the presence of 1 µM CellEvent as described in Figure 2 legend. LysoTracker Deep Red (20 nM final) was added (*T* = 0), and the images were acquired at the indicated time points using the CellInsight NXT HCS platform. LysoTracker Deep Red fluorescence (Cy5 filter set) and florescence area were measured using a Morphology.V4 application. The plots show the changes in spheroid-associated LysoTracker fluorescence analyzed with “R” software (mean ± SD, six spheroids per condition). **(C,D)** Representative images of **(C)** VCaP and **(D)** LNCaP spheroids were acquired on a Zeiss Observer Z1 microscope after 8 h of LysoTracker treatment. Scale bar, 200 µm.

Because CellEvent analysis of apoptosis did not detect any significant effect of 50 nM MLN on LNCaP spheroids, we looked for other vital probes that were more sensitive. We found that spheroid staining with LysoTracker Deep Red was inversely proportional to MLN concentration. When added to the spheroids, the fluorescence of LysoTracker steadily increased and then started to plateau after 8 h of incubation (Figures 3A,B). The plateau level in MLN-pretreated spheroids was maximal in control conditions and decreased with increasing MLN concentration. The staining pattern was complementary to that of CellEvent, with the LysoTracker accumulating in the outer and presumably metabolically active layers of spheroids (Figures 3C,D). This result was unexpected because LysoTrackers are considered to be markers of cytotoxicity, cell senescence and apoptosis (78–80) (Table 2). One possible explanation for this discrepancy is that LysoTracker Deep Red belongs to a different chemical class than LysoTracker Red DND-99, which was used in previous studies (78–80).

Nevertheless, the use of LysoTracker Deep Red allowed for fair quantification of the effect of different doses of MLN with both VCaP and LNCaP spheroids. Similar to CellEvent, LysoTracker Deep Red dye is non-toxic (at tested concentrations <50 nM) and can be used for long-term monitoring of the drug effect in a 3D spheroid model.

### Phenotypic Changes Induced by MLN in Prostate Cancer Spheroids

**Figure 4:**
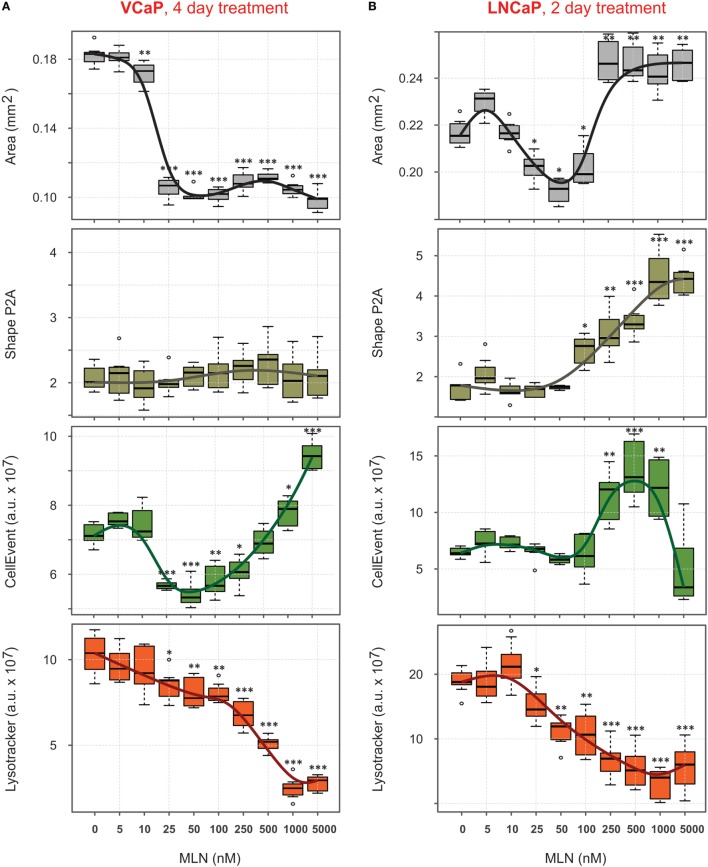
Quantification of phenotypic changes induced by MLN in prostate cancer spheroids. **(A,B)** Preformed spheroids were treated for 4 days [VCaP, **(A)**] or 2 days [LNCaP, **(B)**] with the indicated concentrations of MLN in the presence of 1 µM CellEvent and stained for 8 h with LysoTracker Deep Red as described in Figure 3 legend. Bright field and fluorescence images were acquired with the CellInsight NXT HCS platform and analyzed using the Morphology.V4 application. The box plots created with “R” software show the changes in spheroid phenotype induced by MLN compared with the mean values in the control spheroids (mean ± SD, six spheroids per condition, per plate, *P*-values: ***<0.001, **<0.01, and *<0.05).

The four parameters described earlier, “Area,” “Shape P2A,” “CellEvent,” and “LysoTracker Deep Red,” allowed for high-content monitoring of the drug effect in real-time. This is an important advantage compared with standard endpoint assays, because it reveals dynamic aspects of drug action and provides a flexible choice to measure dose–response curves. Using this approach, the complexity of the MLN effect, which is not obvious in monolayer culture (77), becomes readily dissected (Figures 4A,B). Thus, in VCaP spheroids, the two major outcomes of MLN treatment were (i) spheroid growth arrest with EC50 = 18 nM (Figure 4A, “Area”) and (ii) a cytotoxic effect with EC50 > 500 nM seen by an increase in apoptosis (Figure 4A, “CellEvent”) and a decrease in LysoTracker Deep Red uptake (Figure 4A, “LysoTracker”). A slight decline in total LysoTracker fluorescence at MLN concentration <100 nM most likely reflected the decrease in spheroid size. The quiescent state, characterized by cell cycle arrest and apoptosis inhibition, is easily detected between 25 and 100 nM MLN (Figure 4A). As we previously showed (77), this state is reversible, and removal of the drug results in spheroid regrowth at a normal pace. In the more MLN-sensitive LNCaP spheroids, these two effects could not be separated because of the earlier onset of cytotoxicity manifested by an increase in “CellEvent” (EC50 = 180 nM, Figure 4B) and a decrease in LysoTracker fluorescence (EC50 = 70 nM, Figure 4B). The major outcome of MLN treatment of LNCaP spheroids was spheroid disruption (Figure 4B, “Area”: EC50_2_ = 190 nM, and “Shape P2A”: EC50 = 250 nM). The growth arrest induced by low <100 nM concentrations of MLN was also detected (Figure 4B, “Area”: EC50_1_ = 18 nM), although it was much less defined than in VCaP spheroids.

### Viability Measurement with an ATP-Based Endpoint Assay

**Figure 5:**
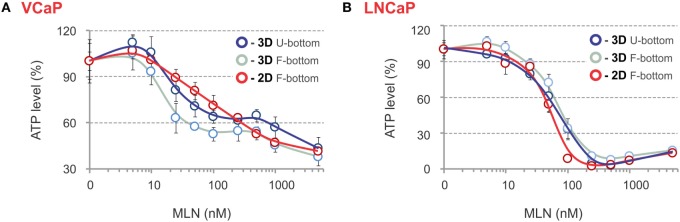
ATP-based endpoint assay to assess the MLN effect. **(A,B)** Spheroids were preformed **(A)** for 7 days with 2,000 VCaP cells and **(B)** for 4 days with 500 LNCaP cells and treated for 4 days (VCaP) or 2 days (LNCaP) with the indicated concentrations of MLN. Then, the spheroids were lysed with ViaLight™ reagent, and luminescence was measured directly in the U-bottomed 96-well NTA plate using a GloMax^®^-Multi Detection System (dark blue curves, 3D U-bottom). Then, the spheroid lysates were transferred into a white plate with a flat transparent bottom (Greiner Bio-One), and luminescence was remeasured (light blue curves, 3D F-bottom). In parallel, VCaP (100,000 cells seeded) and LNCaP (20,000 cells seeded) monolayer cultures in white microtiter plates with a flat transparent bottom (Greiner) were processed similarly with ViaLight™ reagent and analyzed (red curves, 2D F-bottom). The data were normalized to the mean value obtained under control conditions (MLN = 0 nM) and are plotted as the means ± SD (six replicates per condition, per plate).

The phenotypic analysis was complemented by evaluation of spheroid metabolic status using an ATP-based endpoint assay. Usually, this requires spheroid lysis and transfer of the lysate into an opaque-walled flat-bottom plate to measure chemiluminescence. We found that analysis can be performed in the same U-bottom NTA plate. Although this causes an approximate threefold decrease in signal intensity, the high sensitivity and dynamic range of the method ensure very similar robust results independently of the plate (Figures 5A,B). The ATP response curves most closely matched those measured with LysoTracker Deep Red, with the biphasicity of the VCaP response becoming even more obvious (Figure 5A). Biphasic drug dose–response curves have been documented previously and are attributed to the heterogeneity of cell populations within spheroids (83). However, phenotypic analysis of the MLN effect in VCaP spheroids and the results of our previous work (77) suggest the complexity of the drug action mechanism is the prime cause. Notably, ATP measurements in VCaP monolayer culture did not show a biphasic response to MLN (Figure 5A), and additional assays were required to detect the difference in drug effect at low and high concentrations (77). Alternatively, for LNCaP cells, similar MLN dose–response curves were obtained with either spheroids or monolayer culture (Figure 5B).

Collectively, these results show that high-content spheroid monitoring provides information that goes beyond a simple cytotoxicity assay. Thus, despite a unique target [NAE, with an IC50 of approximately 5 nM (87)], inhibition of neddylation by MLN affects multiple cellular processes with different effects on cell viability (77). This explains why MLN toxicity in various cell lines varies significantly. LNCaP cells are 10 times more sensitive than VCaP cells, despite a similar efficiency of neddylation inhibition (EC50 = 8–10 nM, Table 3). Some of these effects can be addressed by high-content monitoring. Our analysis shows that spheroid growth is the most sensitive process affected by MLN (EC50 = 18 nM, Table 3). Because the proposed mechanism of MLN toxicity is proliferation dependent (96), it seems plausible that in VCaP spheroids inhibition of cell proliferation results in protective quiescence [Figure 4A, and Ref (77)], while in the faster-proliferating LNCaP cells, this inhibition is not efficient and does not prevent the onset of apoptosis and cytotoxicity (Figure 4B).

**Table 3:** Quantitative evaluation of MLN effect.

	Growth	Integrity	Apoptosis		Viability	Viability		Nedd8
	Area^a^	P2A^a^	3D-CE^a^	2D-CE^c^	LysoT^a^	3D-ATP^b^	2D-ATP^b^	WB^c^
VCaP	18	n/a	20; >600^d^	500	20; 500^d^	20; >600^d^	500	8
LNCaP	18; 190^d^	250	180	130	70	70	50	10

*MLN EC50 values were estimated from dose–response curves and are expressed in nanomolars. For complex dependency graphs, EC50 was determined as the concentration corresponding to the half change in the monitored parameter between the local maximum and local minimum*.

*P2A, shape P2A; CE, CellEvent; LysoT, LysoTracker Deep Red; WB, western blot*.

*^a^Estimated from the graphs shown in Figure 4*.

*^b^Estimated from the graphs shown in Figure 5*.

*^c^The EC50 values for apoptosis stimulation in monolayer cultures and inhibition of neddylation of cellular proteins were estimated from the data in Ref. (77)*.

*^d^Two EC50 values were tentatively assigned to the dose–response curve*.

### Monitoring the Effect of Chemotherapy Drugs

To further evaluate the method, the effect of four anticancer drugs used for prostate cancer chemotherapy was examined. The following compounds with distinct MOAs were chosen: docetaxel, a microtubule-binding anti-mitotic agent, which is the most commonly used “standard of care” drug for castration-resistant prostate cancer (CRPC) (97, 98); cisplatin, a DNA-crosslinking chemotherapeutic agent, and etoposide, a topoisomerase II inhibitor, which are evaluated in combination therapies for some resistant CRPC phenotypes (98, 99); and ARN-509, a novel antiandrogen for prostate cancer treatment (100), which was recently submitted to the FDA for approval.

The experimental conditions established in the MLN study were used. Four parameters, “Area,” “Shape P2A,” “CellEvent,” and “LysoTracker Deep Red,” were measured to build dose–response curves with VCaP and LNCaP spheroids (Figures S2–S5 in Supplementary Material). At the end of the treatments (2 days for LNCaP and 4 days for VCaP), cell viability within spheroids was measured with a Vialight ATP-based assay, and the resulting dose–response curves were compared with those obtained with monolayer cultures (Figure S6 in Supplementary Material).

The measured EC50 values are summarized in Table 4. The endpoint ATP assay showed that all drugs suppressed cancer cell growth. As often observed in 3D models (101), spheroids showed similar or increased resistance to drug treatments compared with the corresponding 2D cultures (Figure S6 in Supplementary Material).

**Table 4:** Summary of drug responses in VCaP and LNCaP cell lines.

Drug		Growth	Integrity	Apoptosis	Viability	Viability	
	
		Area^a^	Shape P2A^a^	3D-CE^a^	LysoT^a^	3D-ATP^b^	2D-ATP^b^
Cisplatin (μM)	VCaP	5	50	5; 25^c^	50	4; 60^c^	40
	LNCaP	4	30	4; 30^c^	30	30	30

Docetaxel (nM)	VCaP	5	1.5	5	5	3	2
	LNCaP	10	2	15	15	15	2

Etoposide (μM)	VCaP	0.8	5	0.3; 4^c^	1	1	1
	LNCaP	1	n/a	0.8	1.5	2	2

ARN-509 (nM)	VCaP	80	n/a	400	100	100	150
	LNCaP	13	n/a	15	13	n/a	n/a

*EC50 values were estimated from dose–response curves and are expressed in nanomolars (docetaxel and ARN-509) or in micromolars (cisplatin and etoposide). For complex dependency graphs, EC50 was determined as the concentration corresponding to the half change in the monitored parameter between the local maximum and local minimum*.

*CE, CellEvent; LysoT, LysoTracker Deep Red*.

*^a^Estimated from the graphs shown in Figures S2–S5 in Supplementary Material*.

*^b^Estimated from the graphs shown in Figure S6 in Supplementary Material*.

*^c^Two EC50 values were tentatively assigned to the dose–response curve*.

High-content analysis (HCA) confirmed complementarity of the morphometric (“Area” vs “Shape P2A”) as well as the fluorescence (“CellEvent” vs “LysoTracker”) phenotypic parameters for characterization of the drug effects (Figures S2–S5 in Supplementary Material). Compared with the ATP assay, an analysis of the phenotypic changes revealed a more complex effect of the drug action. Thus, similar to MLN, the dose–response curves were not always sigmoidal or monotonic (see, for example, CellEvent apoptosis measurements, Figures S2 and S3 in Supplementary Material), which resulted in more than one EC50 value (Table 4). The sign of the effect could also vary depending on the drug. For instance, docetaxel induced an increase in the “Area” parameter in both VCaP and LNCaP spheroids, while all other drug treatments reduced the spheroid size (Figures S2–S5 in Supplementary Material). Furthermore, the obtained EC50 values differed, sometimes significantly, from those measured with the ATP-endpoint assay and often were smaller.

**Figure 6:**
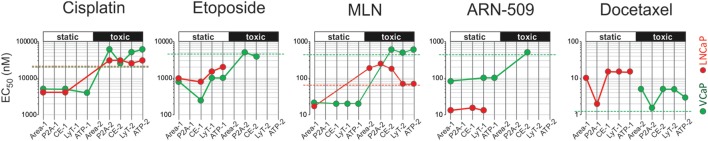
High-content analysis plots for drug response in VCaP and LNCaP spheroids. The plots were constructed using the data in Tables 3 and 4. The EC50 values were estimated from dose–response curves for “Area,” “Shape P2A” (P2A), “CellEvent” (CE), “LysoTracker” (LysoT), and ATP parameters, which were measured in VCaP (green circles) and LNCaP (red circles) spheroids. The increments “−1” and “−2” indicate lower and higher values, respectively. The threshold lines correspond to the drug concentration at which no change in spheroid ATP content was observed within a given treatment time [2 days for LNCaP (red line) and 4 days for VCaP (green line)]. The EC50 values below the threshold were tentatively assigned to the cytostatic drug effect, whereas those above the threshold were assigned to the cytotoxic drug effect.

To summarize the HCA results, we plotted all EC50 values measured for a particular drug on the same diagram (Figure 6). Because up to two EC50s could be observed for each phenotypic parameter, the increments “−1” and “−2” were used to indicate lower and higher value, respectively. For each cell line, we traced a “static” threshold line that corresponded to the drug concentration at which no change in spheroid ATP content was observed within a given treatment time [2 days for LNCaP (red line) and 4 days for VCaP (green line)]. Using these thresholds, the observed EC50 values were tentatively assigned to cytostatic (value below threshold) or cytotoxic (above threshold) drug action. As a result, some treatments appeared mostly cytostatic (etoposide and ARN-509 in both cell lines, docetaxel in LNCaP), whereas others showed a concentration-dependent cytostatic-to-cytotoxic transition (cisplatin and MLN in both cell lines) or purely cytotoxic effect (docetaxel in VCaP) (Figure 6).

**Figure 7:**
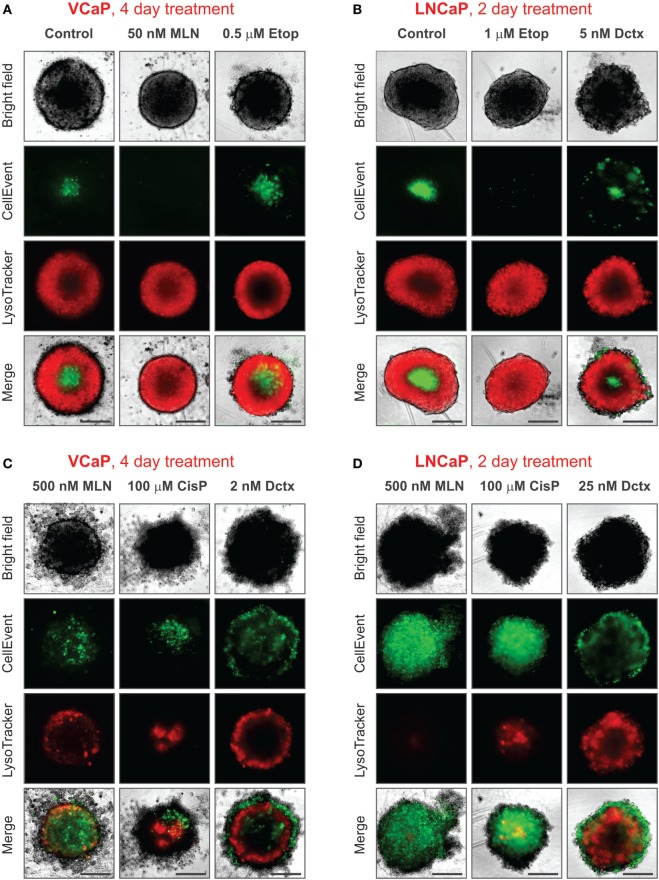
Various phenotypes of drug-treated spheroids. The experimental conditions were the same as described in Figure 3 legend. **(A,B)** Distinct cytostatic phenotypes. **(C,D)** Distinct cytotoxic phenotypes. Scale bar, 200 µm.

It should be noted that conditions underlying spheroid growth arrest were different depending on the drug. Thus, the cytostatic effect of low doses of MLN in VCaP and etoposide in LNCaP spheroids was characterized by stabilization of spheroid size, a slight increase in mean LysoTracker fluorescence and the absence of drug-induced apoptosis (Figures 7A,B). The formation of an apoptotic spheroid core was also completely prevented. As we previously showed for MLN, this rather stable quiescent state resulted from G0/G1 cell cycle arrest and could be reversible (77). By contrast, etoposide treatment of VCaP or docetaxel treatment of LNCaP spheroids was characterized by a significant apoptosis rate (Figures 7A,B). Many apoptotic cells were localized on the periphery and in protruding blebs. This suggests that the apparent cytostatic state was a result of a stationary equilibrium between cell proliferation and expulsion of dying cells, as it was previously described for tissue-derived colospheres and organotypic multicellular spheroids (5). This condition seems transient because extension of docetaxel treatment to 4 days resulted in a significant decline in viability of LNCaP spheroids, similar to that observed in VCaP cells.

Finally, despite the observation that the cytotoxic effects of different drugs shared many common characteristics, i.e., ATP decline, apoptosis, decrease in LysoTracker uptake, and compromised integrity (increase in “Shape P2A”), the corresponding spheroid phenotypes were very distinct (Figures 7C,D). This difference apparently reflects a particular MOA of the drugs. As a result, spheroids formed by different cell lines but treated with the same drug had greater resemblance than spheroids formed by the same cell line but treated with different drugs (see, for example, the characteristic “halo” of apoptotic cells in docetaxel-treated spheroids, Figures 7C,D). In our study, the most striking example of the unique drug effects was the asymmetric unilateral spheroid disruption seen in almost all LNCaP spheroids treated with 500 nM MLN (Figures 2D, 3D and 7D).

## Discussion

Recent technological developments have renewed an interest in spheroid culture as a pertinent model for drug screening. However, although the availability of spheroid-specific consumables and high-content imaging platforms promises to make spheroid analysis easier, it remains far from routine in many laboratories.

We describe a method that allows easy and straightforward generation of uniform multicellular spheroids, their staining with vital dyes, real-time monitoring of the drug effect, and endpoint viability assays to be performed sequentially in the same 96-well microtiter plate. To demonstrate the method performance, we used inexpensive NTA plates (instead of ULA plates) that, nevertheless, ensured robust spheroid generation and analysis. Furthermore, although we used the CellInsight HCS platform, spheroid imaging can also be performed on standard or lens free (92) microscopes and analyzed using open-source tools, e.g., based on MATLAB (39), ImageJ (83), or Fiji (93).

There are several points to consider with the use of Falcon^®^96 Non-Treated Assay Plates (#353910). First, among the various non-treated polystyrene plates we examined, these plates performed the best in spheroid formation. Second, the plates are supplied non-sterile in peel-open medical-style packaging. Although we never had contamination problems when using standard antibiotic-supplemented media (for up to 3 months of spheroid culturing), some antibiotic-sensitive applications, e.g., DNA or siRNA transfection, may require plate sterilization. In our laboratory, a 30-min UV sterilization protocol (102) works well, although sterilization by gas or γ-irradiation can also be used when available. Third, the plates are supplied without lids and can be covered by any lids matching standard 96-well plates, such as those supplied by Thermo (# 5500). Finally, not all cell lines form spheroids in NTA plates. For cell lines, such as PC3, that form only loose aggregates (Figure 1A; Figure S1 in Supplementary Material), supplementation of the culture medium with extracellular matrix components can help promote spheroid assembly (21, 33, 103). For immortalized RWPE-1 prostate cells, which do not form spheroids because they are able to attach to a non-treated polystyrene surface and grow in a monolayer, addition of 10% FBS enables efficient spheroid formation (Figure S1 in Supplementary Material). For other cell lines, such as PNT2 and DU145, the use of ULA plates may be preferable.

When performing long-term spheroid monitoring, special attention must be paid to cell seeding density and growth rate. These two parameters determine spheroid size and total imaging time. The optimal spheroid size depends on the assay requirements, e.g., the presence or not of a hypoxic core and optimal fluorescence staining, but is usually within a 200–500 µm diameter range (21, 31, 83). The size is also limited by the instrument imaging capacity. Thus, with acquisition of one field of view per well (896.6 µm × 896.6 µm for a 10× objective on CellInsight NXT), the diameter of spheroids should not exceed 800 µm. Considering that spheroids are not always perfectly centered within the wells and that spheroids disrupted by drugs may have an irregular shape (Figures 2D and 3D), this size limit may be even smaller (<600 μm).

Furthermore, large spheroids have more significant molecular (nutrient, metabolite, drug, probe, etc.) gradients that can affect the apparent phenotypic outcome of the drug treatment. For example, the CellEvent apoptotic signal produced by the hypoxic core of a fast-growing spheroid (>450 μm in diameter) may be comparable or even greater than that induced by a drug, thus interfering with cytotoxicity measurement. In such cases, analysis of smaller spheroids may be preferable.

Although spheroid size and shape remain the primary parameters for real-time monitoring of a drug effect (21, 38, 39), the use of fluorescent probes provides additional information regarding the mechanism of drug action (20, 23, 47, 48). In principle, the more probes you use, the more information you gather. However, the probes should not interfere with spheroid growth, the drug effect or each other. Here, we show that the use of CellEvent and LysoTracker Deep Red ensures independent real-time monitoring of the MLN effect on spheroid viability and cell apoptosis. Combined with the morphometric analysis and ATP-based endpoint viability assay, this allows for the complex effect of the drug action to be described in a series of EC50 values (Tables 3 and 4; Figure 6).

## Concluding Remarks

In recent years, significant efforts have been made to implement 3D models in drug discovery protocols (1, 101, 104). The hope is that using 3D models will result in more efficient selection of drug candidates and diminish the rate of drug attrition. Indeed, the transition from monolayer culture to a more natural 3D context can significantly alter the response of cancer cells to chemotherapeutics. Generally, cancer cells cultured in 3D systems become more refractory to various treatments due to limited drug penetration and activation of numerous resistance mechanisms, although the opposite has also been observed [see Ref. (101, 104) for review]. Consequently, many reports have focused on the difference in drug cytotoxicity in various cancer models (46, 74, 105–107). Beyond the cytotoxicity issue, 3D models have also been applied to discover molecules that suppress tumor-relevant processes, such as hypoxia-induced drug resistance (23), metastasis-like invasion and cancer cell spreading (21), and cancer-associated fibroblast infiltration (20). Often, analysis of complex phenotypes requires high-content monitoring of multiple parameters. For example, HCA coupled with an advanced image treatment has been successfully performed in a semi-automated manner on heterogeneous matrix-embedded 3D cultures (108–110). Such analysis is significantly simplified when an array of uniform spheroids that allows reproducible detection of fine changes in specific cellular phenotype is used (20, 23, 47–49). Thus, the effect of drugs on multiple cellular processes, such as mitochondria function, apoptosis, and cell homeostasis, can be followed using specific fluorescent probes (Table 2) with a high level of confidence.

In this report, we demonstrate that simultaneous monitoring of two morphometric and two fluorescence parameters permits clear discrimination between cytostatic and cytotoxic drug effects. This may be particularly pertinent for preclinical evaluation of drug effects, because cytostatic conditions are often implicated in tumor dormancy, drug resistance, and development of more aggressive phenotypes (111, 112). Compared with monolayer cultures, monitoring of 3D phenotypic parameters, such as spheroid size, shape, and internal organization report on “macroscopic” effects of drug treatment. Numerous studies have reported that different drugs induce quite distinct spheroid phenotypes (23, 47, 48). We observed that docetaxel and MLN produced characteristic changes in spheroid morphology that likely reflect a particular mechanism of drug action. Detailed characterization of a drug-induced phenotype may therefore help in evaluating the outcomes of drug actions on a multicellular/tissue level. In this context, and for future development of the method, 3D-confocal imaging can be used to enable efficient 3D reconstruction of spheroid phenotypes *via* z-stacking and subsequent 3D convolution (47, 113). We believe that once fully validated in a wider range of conditions the method described here could be particularly valuable for phenotype-based drug discovery (114–116).

## Author Contributions

FM and PO performed most of the data acquisition and analysis and participated in designing the experiments and writing the manuscript. AR and VH performed some of the experiments and participated in data analysis and the writing of the manuscript. XG provided conceptual input and participated in data analysis and the writing of the manuscript. MB designed and supervised the experiments, analyzed the data, and wrote the manuscript.

## Conflict of Interest Statement

The authors declare that the research was conducted in the absence of any commercial or financial relationships that could be construed as a potential conflict of interest.

## Supplementary Material

Video S1 VCaP spheroid assembly.
