# Supplementary material for: High-Content Monitoring of Drug Effects in a 3D Spheroid Model
Source: Front Oncol. 2017 Dec 11;7:293. doi: 10.3389/fonc.2017.00293 (PMC5732143; doi:10.3389/fonc.2017.00293)
Supplement: Supplementary file 1 [file Presentation_1.PDF]

SUPPLEMENTARY INFORMATION

High-content monitoring of drug effects in a 3D spheroid model

Frédérique Mittler[1], Patricia Obeid[1], Anastasia V. Rulina[1,2], Vincent Haguet[1], Xavier Gidrol[1], and Maxim Y. Balakirev[1]**

[1]Univ. Grenoble Alpes, CEA, INSERM, BIG, BGE, F-38000 Grenoble, France
[2]Univ. Lyon 1, ENS de Lyon, INSERM, CNRS, CIRI, F-69007 Lyon, France
*Correspondence: Xavier Gidrol, xavier.gidrol@cea.fr
*Correspondence: Maxim Balakirev, maxim.balakirev@cea.fr

TABLE OF CONTENTS

Pages

Supplementary Table S1.. 2

Supplementary Figures S1-S6... 3-8

Drug	M.W.	Target[1]	Drug final concentration (nM)									
**MLN4924**	444	NAE	0	5	10	25	50	100	250	500	1000	5000
**ARN509**	477	AR	0	5	10	25	50	100	250	500	1000	5000
**Docetaxel**	808	Microtubules	0	0.2	1	2	5	10	25	50	100	500
**Etoposide**	589	Topoisomerase II	0	10	50	250	500	1000	2500	5000	10000	20000
**Cisplatin**	300	DNA	0	10	50	250	1000	5000	10000	25000	50000	100000

Supplementary Table S1. **Drugs used in the study.** Drug stock solutions were prepared in DMSO, with the exception of cisplatin, for which DMF was used. (1) Abbreviation: NAE- Nedd8 activating enzyme; AR-androgen receptor.

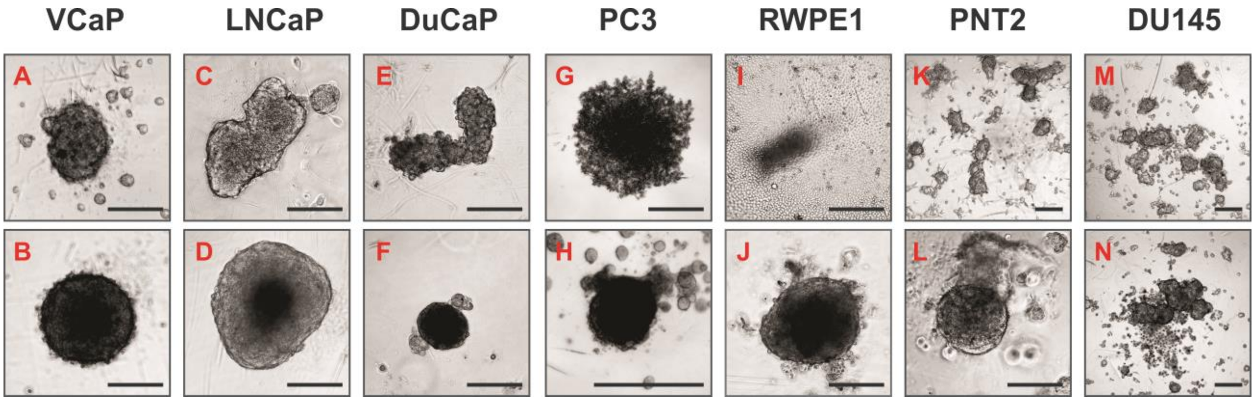

Supplementary Figure S1. **Spheroid formation by various prostate cell lines in U-bottomed NTA plates.** **(A,B)** The aggregates formed by 2000 VCaP cells cultured for 4 days in DMEM containing 10% FBS (**B**) or 10% charcoal-stripped FBS (**A**). **(C,D)** The aggregates formed by 500 LNCaP cells cultured for 9 days in RPMI containing 10% FBS (**D**) or 10% charcoal-stripped FBS (**C**). **(E,F)** The aggregates formed by 2000 DuCaP cells cultured for 9 days in DMEM containing 10% FBS (**F**) or in RPMI containing 10% FBS (**E**). **(G,H)** The aggregates formed by 500 PC3 cells cultured for 5 days in RPMI containing 10% FBS and supplemented (**H**) or not (**G**) with 2.5% Matrigel. **(I,J)** The monolayer and aggregates formed by 2000 RWPE1 cells cultured for 4 days in K-SFM containing 0.05 mg/mL BPE, 5 ng/mL EGF and supplemented (**J**) or not (**I**) with 10% FBS. **(K,L)** The aggregates formed by 1000 PNT2 cells cultured for 7 days in RPMI containing 10% FBS and supplemented (**L**) or not (**K**) with 0.5% Matrigel. **(M,N)** The aggregates formed by 1000 PNT2 cells cultured for 7 days in RPMI containing 10% FBS and supplemented (**L**) or not (**K**) with 0.5% Matrigel.

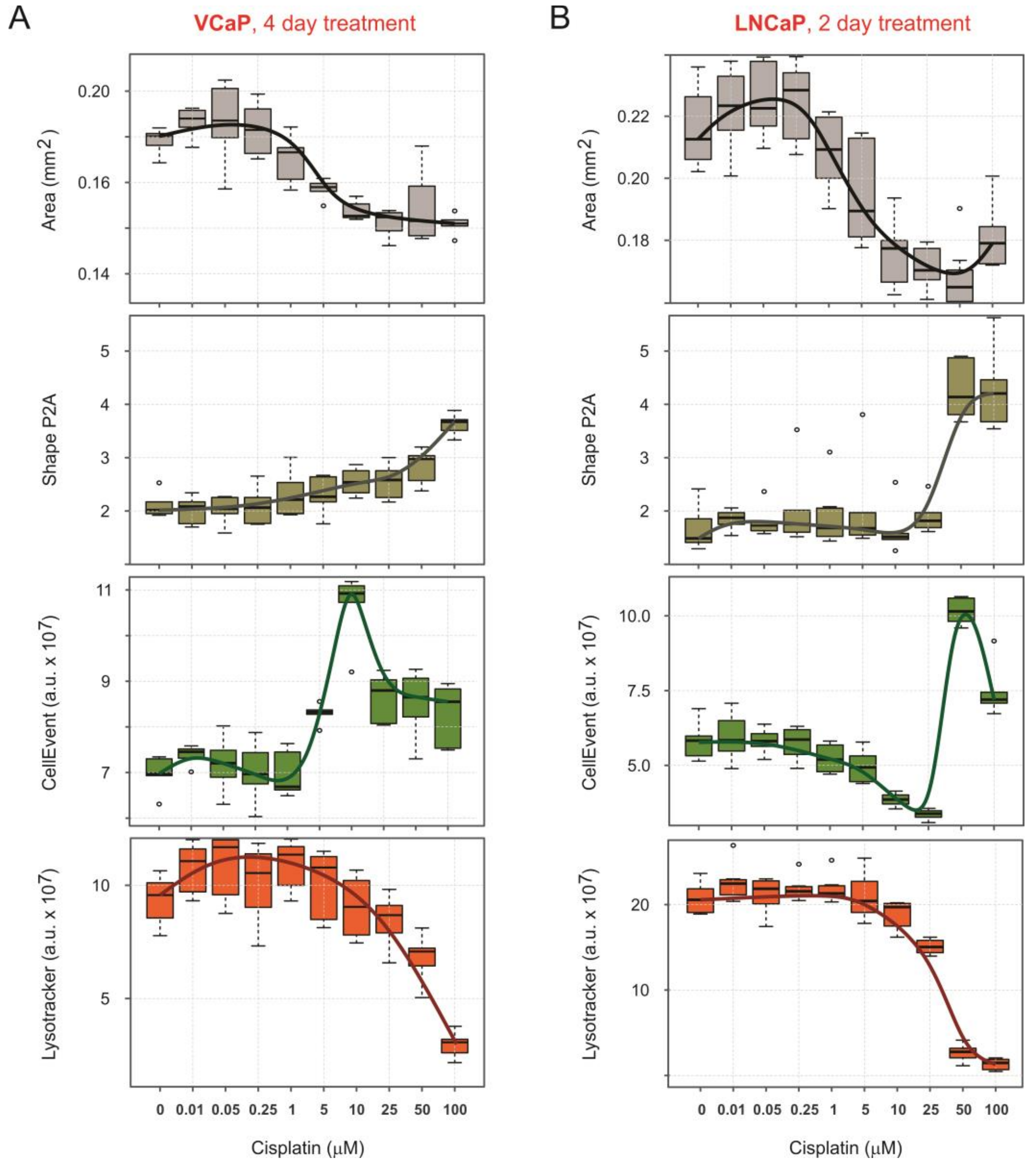

Supplementary Figure S2. Quantification of phenotypic changes induced by cisplatin in prostate cancer spheroids. (A,B) Pre-formed spheroids were treated for 4 days (VCaP, **(A)**) or 2 days (LNCaP, **(B)**) with indicated concentrations of cisplatin in the presence of 1 μM CellEvent and stained for 8 hours with LysoTracker Deep Red as described in **Figure 4** legend.

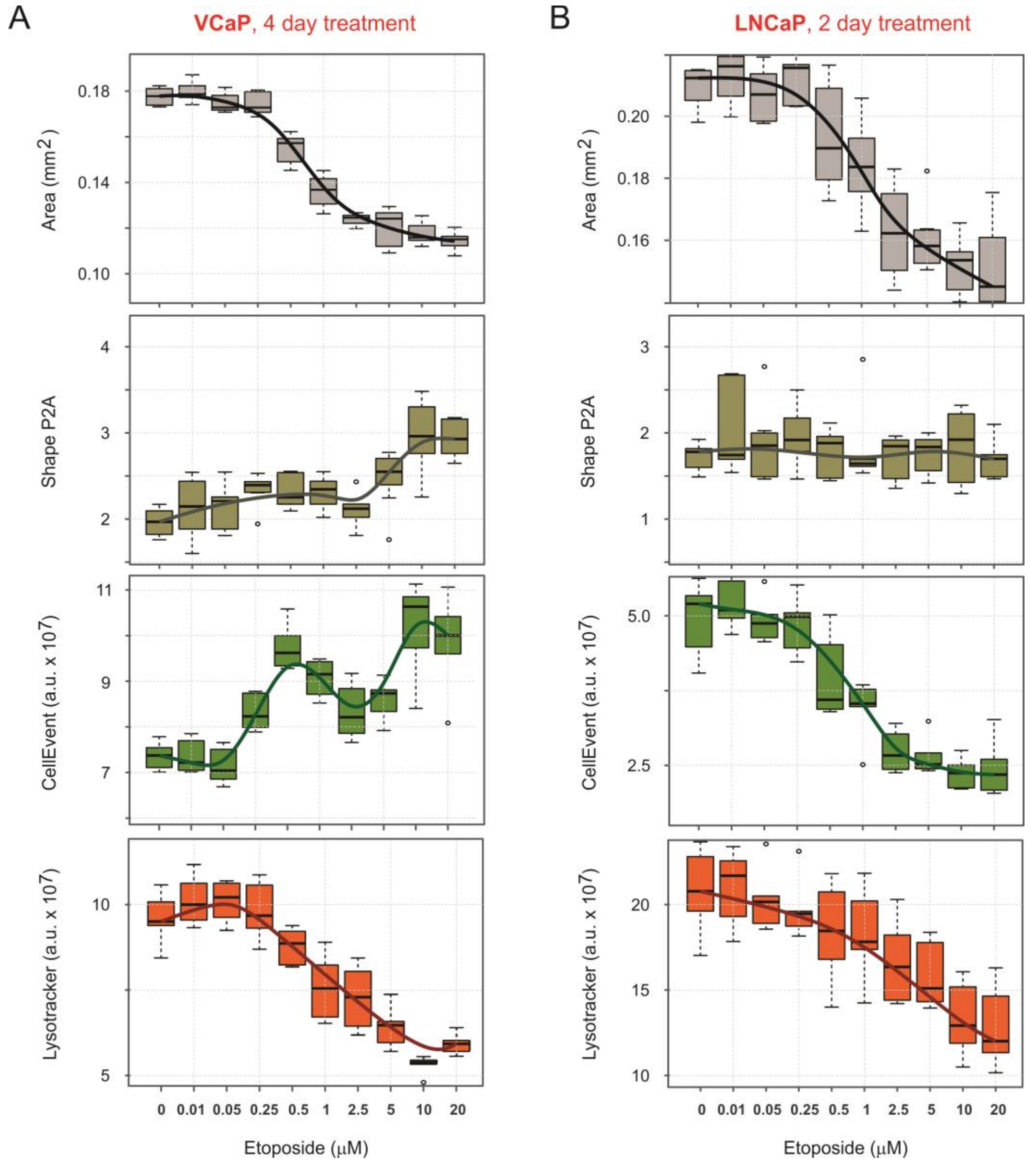

Supplementary Figure S3. Quantification of phenotypic changes induced by etoposide in prostate cancer spheroids. (A,B) Pre-formed spheroids were treated for 4 days (VCaP, **(A)**) or 2 days (LNCaP, **(B)**) with indicated concentrations of etoposide in the presence of 1 μM CellEvent and stained for 8 hours with LysoTracker Deep Red as described in **Figure 4** legend.

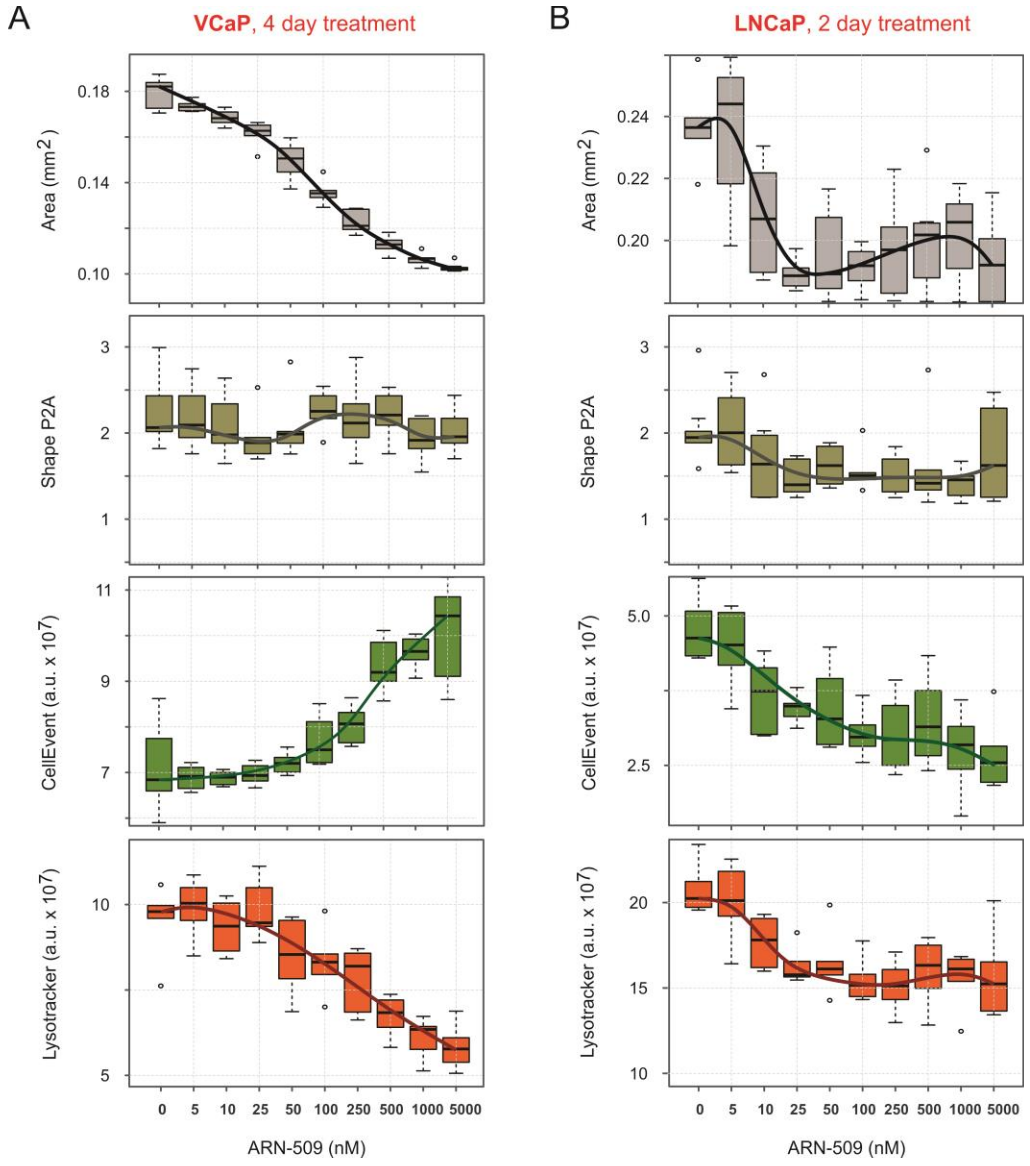

Supplementary Figure S4. Quantification of phenotypic changes induced by ARN-509 in prostate cancer spheroids. (A,B) Pre-formed spheroids were treated for 4 days (VCaP, **(A)**) or 2 days (LNCaP, **(B)**) with indicated concentrations of ARN-509 in the presence of 1 µM CellEvent and stained for 8 hours with LysoTracker Deep Red as described in **Figure 4** legend.

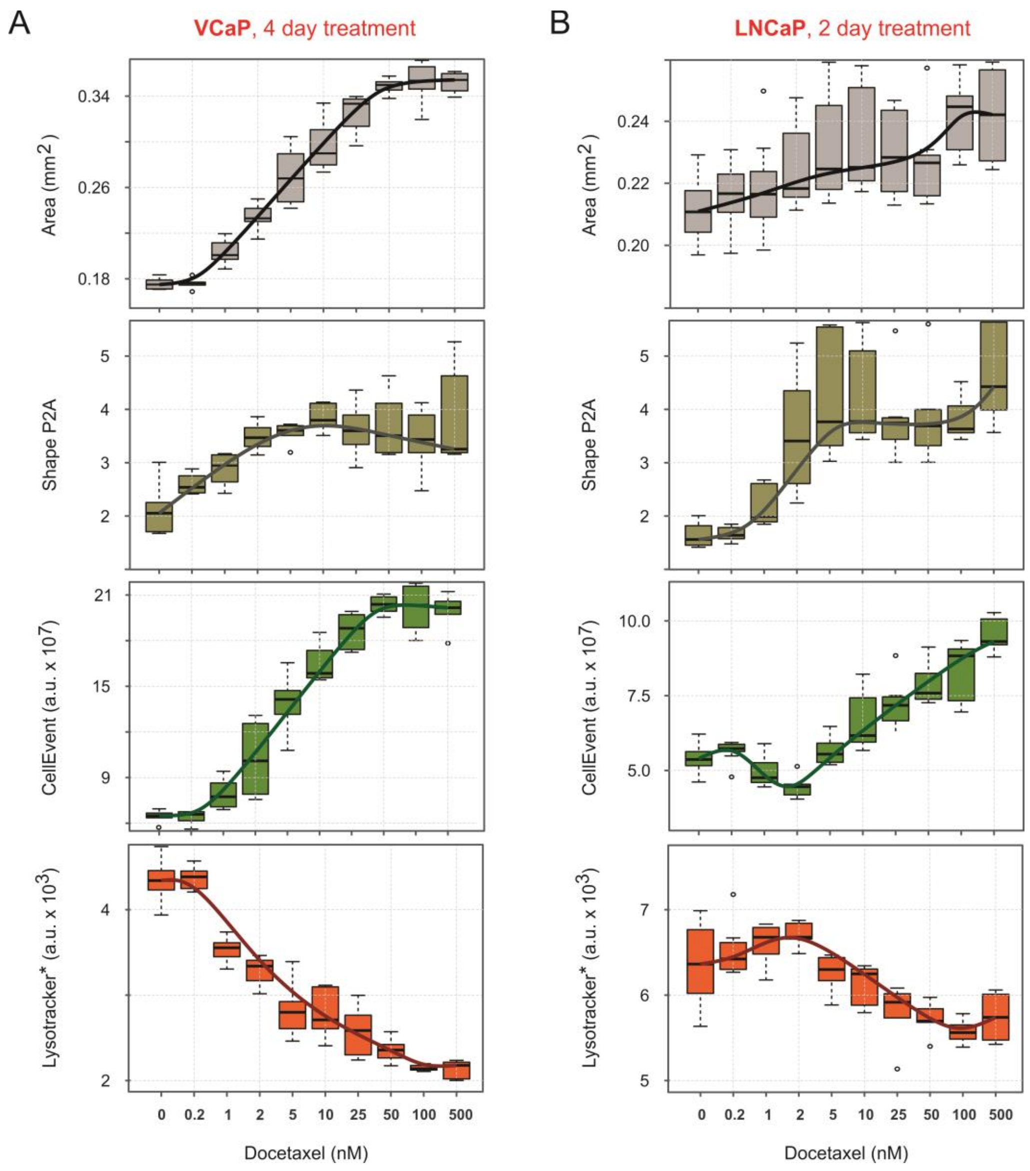

Supplementary Figure S5. Quantification of phenotypic changes induced by docetaxel in prostate cancer spheroids. (A,B) Pre-formed spheroids were treated for 4 days (VCaP, **(A)**) or 2 days (LNCaP, **(B)**) with indicated concentrations of docetaxel in the presence of 1 µM CellEvent and stained for 8 hours with LysoTracker Deep Red as described in **Figure 4** legend.

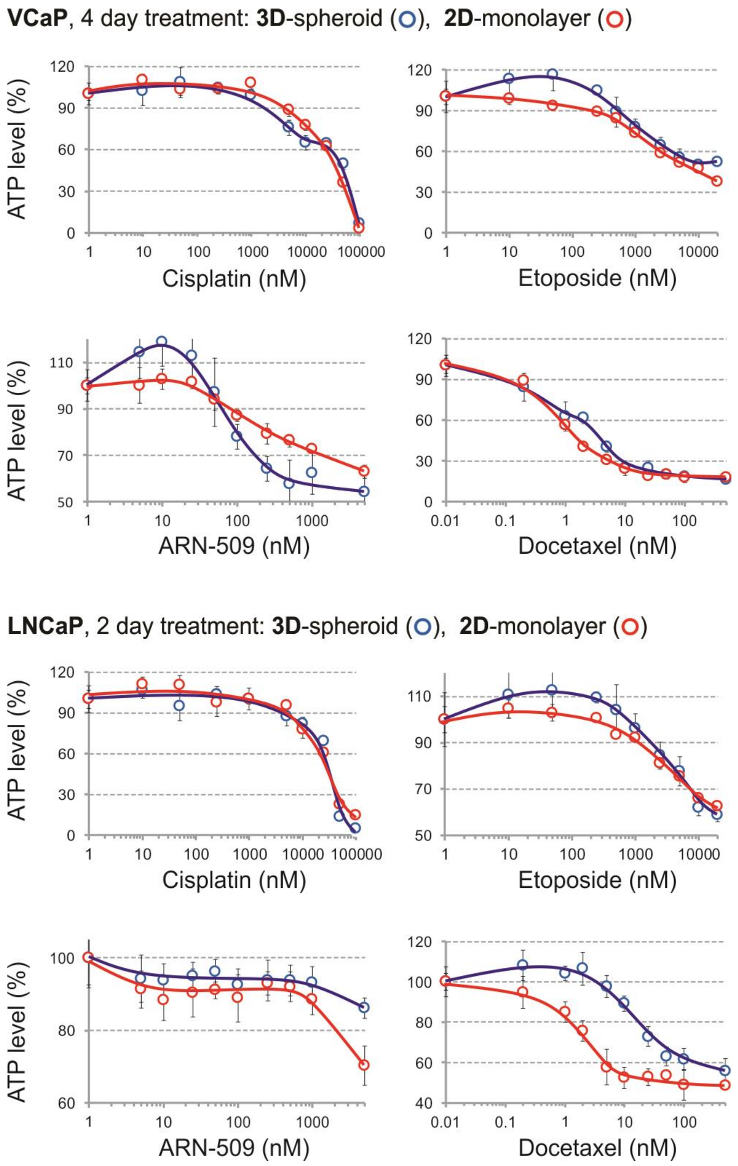

Supplementary Figure S6. ATP-based endpoint assay of the drug effect. (A,B) Spheroids were pre-formed for 7 days with 2000 VCaP cells **(A)** and for 4 days with 500 LNCaP cells **(B)**, and treated for 4 days (VCaP) or 2 days (LNCaP) with indicated drugs. After that, the spheroids were lysed with ViaLight™ reagent, and luminescence was measured as described in **Figure 5** legend.
